# Brain Activity Characteristics of Patients With Disorders of Consciousness in the EEG Resting State Paradigm: A Review

**DOI:** 10.3389/fnsys.2022.654541

**Published:** 2022-05-27

**Authors:** Anna Duszyk-Bogorodzka, Magdalena Zieleniewska, Kamila Jankowiak-Siuda

**Affiliations:** ^1^Behavioural Neuroscience Lab, Institute of Psychology, SWPS University of Social Sciences and Humanities, Warsaw, Poland; ^2^Faculty of Physics, University of Warsaw, Warsaw, Poland

**Keywords:** electroencephalography, EEG connectivity, resting state EEG, disorders of consciousness, vegetative state, unresponsive wakefulness syndrome, minimally consciousness state

## Abstract

The assessment of the level of consciousness in disorders of consciousness (DoC) is still one of the most challenging problems in contemporary medicine. Nevertheless, based on the multitude of studies conducted over the last 20 years on resting states based on electroencephalography (EEG) in DoC, it is possible to outline the brain activity profiles related to both patients without preserved consciousness and minimally conscious ones. In the case of patients without preserved consciousness, the dominance of low, mostly delta, frequency, and the marginalization of the higher frequencies were observed, both in terms of the global power of brain activity and in functional connectivity patterns. In turn, the minimally conscious patients revealed the opposite brain activity pattern—the characteristics of higher frequency bands were preserved both in global power and in functional long-distance connections. In this short review, we summarize the state of the art of EEG-based research in the resting state paradigm, in the context of providing potential support to the traditional clinical assessment of the level of consciousness.

## 1. Introduction

After severe brain damage, some patients suffer from disorders of consciousness (DoC), such as coma, unresponsive wakefulness syndrome (UWS), or minimally conscious state (MCS). In patients with UWS, no behavioral signs of consciousness are observed (Giacino et al., 2004). Those in MCS manifest volitional reactions, such as visual pursuit, pain localization (MCS), and/or command following movements (MCS+) but no communication is possible (Bruno et al., 2011). However, as Gosseries et al. (2014) and Thibaut et al. (2021) pointed out, some patients with UWS have brain activity patterns characteristic of MCS but no traces of consciousness are observed in their behavior. These patients are named non-behavioral MCS (MCS *). Establishing communication and/or regaining the skill of functional use of an object indicates the emergence of MCS (eMCS) and recovery of consciousness (Giacino et al., 2002, 2004). Electroencephalography (EEG) is a common diagnostic technique in routine clinical neurological management. Due to its mobility and wide availability, it offers an indication of a patient's brain functioning, allowing for diagnosis and regular monitoring of their condition. In the case of patients with DoC, brain activity shows huge inter-patient variability. Following a brain injury, EEG can be altered and many abnormal patterns can be observed, e.g., diffuse slowing of activity, polymorphic focal delta rhythm over damaged regions (Brenner, 2005), asymmetries related to brain damage, burst-suppression pattern, and epileptiform activity (Lehembre et al., 2012b). The clearest way to show the presence of consciousness is to use active experimental procedures, where a patient's direct engagement in following instructions is required. However, in the case of patients with DoC, this paradigm has several limitations, including the fluctuation of attention, aphasia, or sensory and motor deficits. These limitations are not relevant in the resting-state EEG analysis, which requires only maintaining an arousal state, without a necessity for language comprehension or patient's active cooperation. The application of resting-state EEG analysis makes it possible to determine the electrical activity of the awake brain in the absence of tasks and instructions (Bai et al., 2017).

The resting state networks were first described in 1995. Using functional magnetic resonance imaging (fMRI), it has been shown that the brains of people who were not given any task to do experienced slow and synchronous changes in sensomotoric regions of the brain that showed so-called “connectivity” relation to one's activities while resting (Biswal et al., 1995). Similar patterns of resting state coherence have been documented in other brain areas, called the default mode network (DMN), and these brain regions are involved in self-referential thinking, emotional processing, and recalling memories (Raichle et al., 2001; Raichle, 2015; Shen, 2015). Using positron emission tomography (PET) and fMRI techniques, many other brain functional networks have been demonstrated at rest to show coherent fluctuations at low frequency (0.01–0.1 Hz), related to active sensory processing (Fox et al., 2005; Damoiseaux et al., 2006). However, it is indicated that the fMRI blood-oxygen-level dependent (BOLD) response may represent the summed neuronal activity observable with EEG signal (Whitman et al., 2013). Brain connectivity can be subdivided into structural and functional connectivity. The former focuses on neuroanatomical links between particular brain structures, the latter reflects the statistical dependencies between the activity of spatially distinct brain areas (Friston, 2011). Functional connectivity is usually measured by correlation, coherence, and information theory (Cao et al., 2021). While functional connectivity can be assessed based on BOLD and EEG signals, structural connections cannot be revealed by the EEG technique. Usually, functional connections are quantified either in frequency or time domain, in particular EEG bands, such as δ, θ, α, β, and γ or for range of the EEG signal, creating so-called EEG networks.

The analyses derived from the resting-state EEG are related to the fundamental state of the brain and seem to help monitor the patient's state of consciousness (Sitt et al., 2014; Bai et al., 2017, 2021; Sebastiano et al., 2021; Wutzl et al., 2021). Owing to the properties of the EEG measurement itself, the indices obtained reflect other aspects of brain functioning, e.g., the frequencies of characteristic rhythms. The most common approaches to the analysis of resting-state activity include the assessment of spectral power, signal complexity, and functional connectivity. The use of resting state EEG in DoC should make it possible to assess the severity of brain damage, bringing us closer to a prognosis about the patient's further condition and understanding the processes acting between different states of consciousness. However, the heterogeneity of patients with DoC and the current means of application of analytical algorithms to resting EEG signals significantly impede data interpretation.

Therefore, the aim of this study is to summarize and discuss EEG resting data based on 38 studies related to the abovementioned entities. As well as outlining aspects of EEG activity that are crucial to the differentiation between unconscious and minimally conscious patients, we also show which of them have prognostic properties.

## 2. Method: Search Strategy

**Figure 1:**
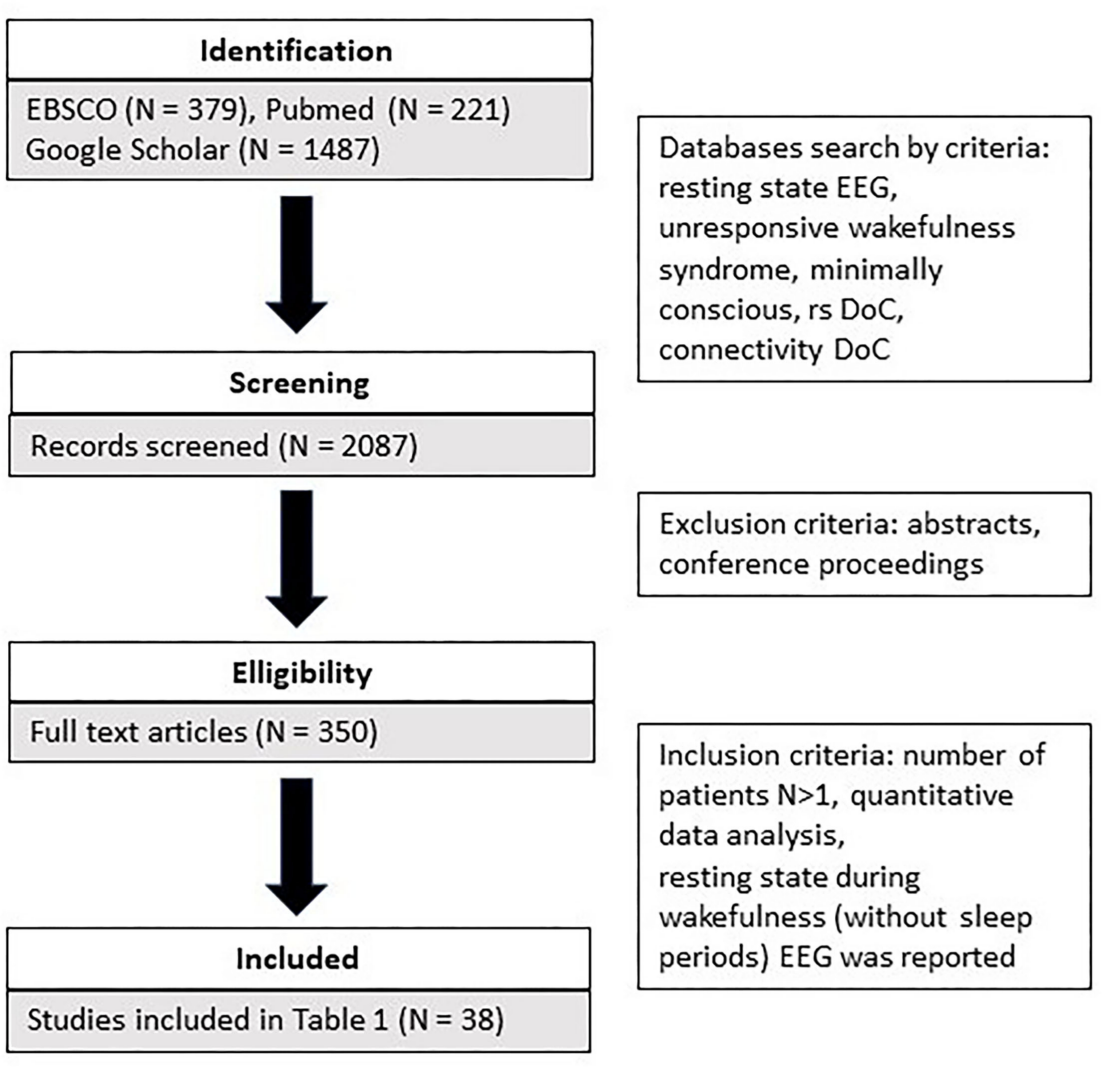
The flowchart of the selection of articles.

The search covered three literature databases (EBSCO, Pubmed, Google Scholar). The following keywords were used as search criteria: resting state EEG and unresponsive wakefulness syndrome and minimally conscious, rs DoC, connectivity DoC. In addition, we identified additional references from those provided in the retrieved articles. A total of 2,081 articles were identified (refer to Figure 1). Subsequently, we screened the retrieved articles for the following inclusion criteria: (a) they were peer-reviewed or conference proceedings and abstracts; b) more than one patient participated in the study; c) only quantitative data analysis was used; d) resting state during wakefulness (without sleep periods) EEG was reported. A detailed comparison of all reviewed studies is summarized in Table 1.

**Table 1:** Summary of studies based on resting-state EEG.

**Objectives**	**References**	**Patients**	**Method**	**Global results**	**δ**	**θ**	**α**	**β**	**γ**
Spectral	Coleman et al., 2005	6 UWS, 4 MCS	Power ratio index	UWS>MCS					
characteristics	Leon-Carrion et al., 2008	7 MCS, 9 SND	Power spectrum, Loreta		MCS>SND	MCS>SND		MCS < SDN	
	Babiloni et al., 2009	50 UWS, 30 HC	Power spectrum, Loreta				UWS < HC, UWS-REC>NON-REC		
	Lehembre et al., 2012a	10 UWS, 21 MCS	Power spectrum		UWS>MCS	UWS < MCS	UWS < MCS		
	Lechinger et al., 2013	8 VS, 9 MCS, 14 HC	Power spectrum		UWS>HC	UWS>HC	UWS, MCS < HC		
	Chennu et al., 2014	13 VS, 19 MCS, 26 HC	Power spectrum - power contribiution		UWS, MCS>HC		UWS, MCS < HC	UWS, MCS < HC	
	Sitt et al., 2014	75 UWS, 68 MCS, 24 CS, 14 HC	Spectral power		UWS>MCS, CS	UWS < MCS, CS	UWS < MCS, CS	UWS, MCS < CS	
	Varotto et al., 2014	18 UWS, 10 HC	Power spectrum		DoC>HC	DoC < HC	DoC < HC	DoC < HC	
	Naro et al., 2016	6 UWS, 7 MCS, 10 HC	Source power (Loreta)		DoC < HC; UWS>MCS	DoC < HC; UWS < MCS	DoC < HC; UWS < MCS	DoC < HC;	DoC < HC; UWS < MCS
	Piarulli et al., 2016	6 UWS, 6 MCS	Spectral power		UWS>MCS	UWS < MCS	UWS < MCS	UWS < MCS	
	Schorr et al., 2016	58 UWS, 15 MCS, 24 HC	Spectral power		DoC>HC		DoC < HC	DoC < HC	
	van den Brink et al., 2018	16 DoC, 16 HC	Spectral amplitude		DoC < HC	DoC < HC	DoC>HC	DoC>HC	
	Naro et al., 2018	17 UWS, 15 MCS	Spectral power		UWS>MCS		UWS>MCS		
	Stefan et al., 2018	51 UWS, 11 MCS	Spectral power		UWS>MCS		UWS < MCS		
	Lutkenhoff et al., 2019	37 UWS, 24 MCS	Spectral power	UWS < MCS	UWS>MCS-	UWS < MCS	UWS, MCS+ < MCS-; UWS>MCS+		MCS+ < MCS-
	Bai et al., 2021	37 UWS, 25 MCS, 25 HC	Spectral power				MCS-V>MCS-NV	MCS-M>MCS-NM	
	Thibaut et al., 2021	11 UWS, 15 MCS^*^, 54 MCS, 33 HC	Spectral power		UWS>MCS^*^	UWS < MCS^*^	UWS < MCS^*^		
	Lei et al., 2022	19 UWS, 21 MCS	Relative wavelet energy		UWS>MCS		UWS < MCS	UWS < MCS, EMCS	
Signal complexity	Schnakers et al., 2008	16 Coma, 13 UWS, 13 EMCS, 30 MCS	Bispectral index	UWS < MCS					
	Sarà and Pistoia, 2010	10 HC, 10 DoC	Approximate entropy	DoC < HC					
	Gosseries et al., 2011	6 Coma, 24 UWS, 26 MCS, 16 HC	Spectral entropy, State entropy, Response entropy	UWS, MCS < HC; UWS < MCS					
	Sarà et al., 2011	38 UWS, 40 HC	Approximate entropy	UWS < HC					
	Wu et al., 2011a	21 UWS, 16 MCS, 30 HC	Lempel-Ziv complexity (LZC), Approximate Entropy (AE), Cross-approximate entropy (CAE)	UWS, MCS < HC (LZC, AE, CAE)					
	Wu et al., 2011b	30 UWS, 20 MCS, 30 HC	Approximate entropy (AC), Cross-approximate entropy (CAE)	UWS < MCS, HC (CAE)					
	King et al., 2013	75 UWS, 68 MCS, 24 CS, 14 HC	Permutation entropy	UWS < MCS, CS					
	Sitt et al., 2014	75 UWS, 68 MCS, 24 CS, 14 HC	Kolmogorov-Chaitin complexity (KC), Permutation entropy (PE), Spectral entropy (SE)	UWS < MCS (KC), UWS < CS, MCS (SE)		UWS < MCS, CS (PE)			
	Piarulli et al., 2016	6 UWS, 6 MCS	Spectral entropy (SE), Wavelet decomposition of spectral entropy time-courses (TC)	UWS < MCS (SE); UWS < MCS (TC)					
	Stefan et al., 2018	51 UWS, 11 MCS	Approximate Entropy, Permutation Entropy (PEn)	ApEn: UWS < MCS			PEn: UWS < MCS		
	Lee et al., 2019a	30 isoflurane anesthesia, 15 ketamine anesthesia, 15 MCS, 27 UWS, 73 HC	Phase lag entropy (PLE): mean values and topographic similarities	mean PLE: UWS < MCS, topographic similarity of PLE: MCS, UWS < HC					
	Lei et al., 2022	19 UWS, 21 MCS	Approximate Entropy (AE), Sample Entropy (SA), Lempel-Ziv Complexity (LZC)	UWS < MCS (AE, SA, LZC); UWS < EMCS (AE, SA, LZC); MCS < EMCS (AE, SA, LZC)					
Functional	Pollonini et al., 2010	7 MCS, 9 SND	Coherence (C), Granger causality (GC)	MCS < SDN (C)	MCS < SDN (C); MCS>SND (GC)	MCS < SDN (C)	MCS < SDN (C)	MCS < SDN (C); MCS < SDN (GC)	
connectivity	Fingelkurts et al., 2012	14 UWS, 7 MCS, 5 HC	Operational Architectonics	UWS, MCS < HC; UWS < MCS				UWS < HC	
networks	Lehembre et al., 2012a	10 UWS, 21 MCS	Coherency (C), Imaginary part of coherency (IC), Phase Lag Index (PLI)		UWS < MCS (IC)	UWS < MCS (IC, PLI)	UWS < MCS (IC, PLI)		
	Leon-Carrion et al., 2012	7 MCS, 9 SND	Coherence (C), Granger causality	MCS < SND (C)	MCS < SND (C)	MCS < SND (C)	MCS < SND (C)	MCS < SND (C)	MCS < SND (C)
	King et al., 2013	75 UWS, 68 MCS, 24 CS, 14 HC	weighted symbolic mutual information (wSMI)	UWS < MCS, CS, HC					
	Cavinato et al., 2014	10 USW, 16 MCS, 15 HC	Coherence			UWS>MCS, HC	MCS, HC>UWS		
	Marinazzo et al., 2014	11 UWS, 10 MCS, 5 EMCS, 10 HC	Multivariate Granger Causality, Transfer entropy	UWS>MCS, EMCS, HC (TE); MCS>EMCS, HC; EMCS>HC					
	Höller et al., 2014	27 UWS, 22 MCS, 23 HC	44 biomarkers from resting EEG and machine learning		UWS>MCS	MCS>UWS	MCS>UWS	UWS>MCS	
	Sitt et al., 2014	68 MCS, 75 VS, 24 CS, 14 HC	Phase-locking index (PLI), Weighted symbolic mutual information (wSMI)		UWS>MCS (PLI)	UWS < MCS (wSMI)	UWS < MCS (wSMI)		
	Chennu et al., 2014	13 UWS, 19 MCS, 26 HC	Debiased weighted phase lag index		UWS, MCS>HC	UWS, MCS>HC	UWS, MCS < HC,		
	Varotto et al., 2014	18 UWS, 10 HC	Partial directed coherence		DoC < HC		DoC>HC		
	Schorr et al., 2016	58 UWS, 15 MCS, 24 HC	Coherence			MCS < HC	UWS, MCS < HC	UWS, MCS < HC	
	Chennu et al., 2017	23 UWS, 17 MCS-, 49 MCS+, 11 EMCS, 4 LIS, 26 HC	Debiased weighted phase lag index		MCS->MCS+		UWS < MCS-		
	Engemann et al., 2018	148 UWS, 179 MCS, 66 HC	Weighted symbolic mutual information			UWS≠MCS			
	Bareham et al., 2018	4 DoC, longitudinal study	Debiased weighted phase lag index		MCS-> MCS		UWS < MCS-		
	van den Brink et al., 2018	16 DoC patients, 16 HC	Correlation of orthogonalized amplitude envelopes		DoC>HC	DoC>HC	DoC < HC		
	Naro et al., 2018	17 UWS, 15 MCS	Debiased weighted phase lag index				UWS < MCS		UWS < MCS; UWS < MCS (in time-course)
	Stefan et al., 2018	51 UWS, 11 MCS	Coherence (C), Weighted symbolic mutual information (wSMI)				UWS>MCS (C)	UWS>MCS (C)	
	Rizkallah et al., 2019	9 UWS, 17 MCS-, 29 MCS+, 6 EMCS, 21 HC	Dynamic functional networks –segregation (C) and integration (P)	MCS- < HC (P)	UWS, MCS < HC (C, P)	EMCS, MCS, UWS<HC (C); MCS, UWS<HC; MCS−, UWS<EMCS; MCS+>MCS−(P)	EMCS, MCS+<HC (P)	MCS+ < HC (P)	UWS, MCS<HC; MCS+<EMCS (P)
	Cai et al., 2020	35 UWS, 19 MCS, 23 HC	Multiplex graph metrics, Multiplex clustering coefficient (MCC), Multiplex participation coefficient (MPC)	UWS>MCS, HC; MCS>HC; (MPC); UWS < MCS, HC (MCC)					
	Bai et al., 2021	37 UWS, 25 MCS, 25 HC	Time-delay embedded Hidden Markov Model (TDE-HMM), Coherence (C)	DoC < HC; MCS < UWS (state dynamics);			DoC < HC (C)		
	Naro et al., 2021	17 UWS, 15 MCS	Multiplexand multilayer network metrics (network topology, NT; multiplex network heterogeneity, MNH)	UWS < MCS (NT)	UWS>MCS (MNH)	UWS>MCS (MNH)	UWS < MCS (MNH)	UWS < MCS (MNH)	
	Thibaut et al., 2021	11 UWS, 15 MCS^*^, 54 MCS, 33 HC	Debiased weighted phase index		UWS>MCS^*^	MCS^*^ < MCS	UWS < MCS^*^, MCS, HC; MCS^*^ < MCS; MCS < HC		

*DoC, disorders of consciousness; HC, healthy controls; CS, conscious patients; SND, severe neurocognitive disorders; LIS, locked-in syndrome; MCS, minimally conscious state; MCS-V/MCS-NV, presence of purposeful/no purposeful visual responses; MCS-M/MCS-NM presence of purposeful/no purposeful motor responses; EMCS, emergence from MCS; UWS, vegetative state; REC/NON-REC, recovery/non-recovery patient*.

## 3. Spectral Characteristics of EEG in DoC

**Figure 2:**
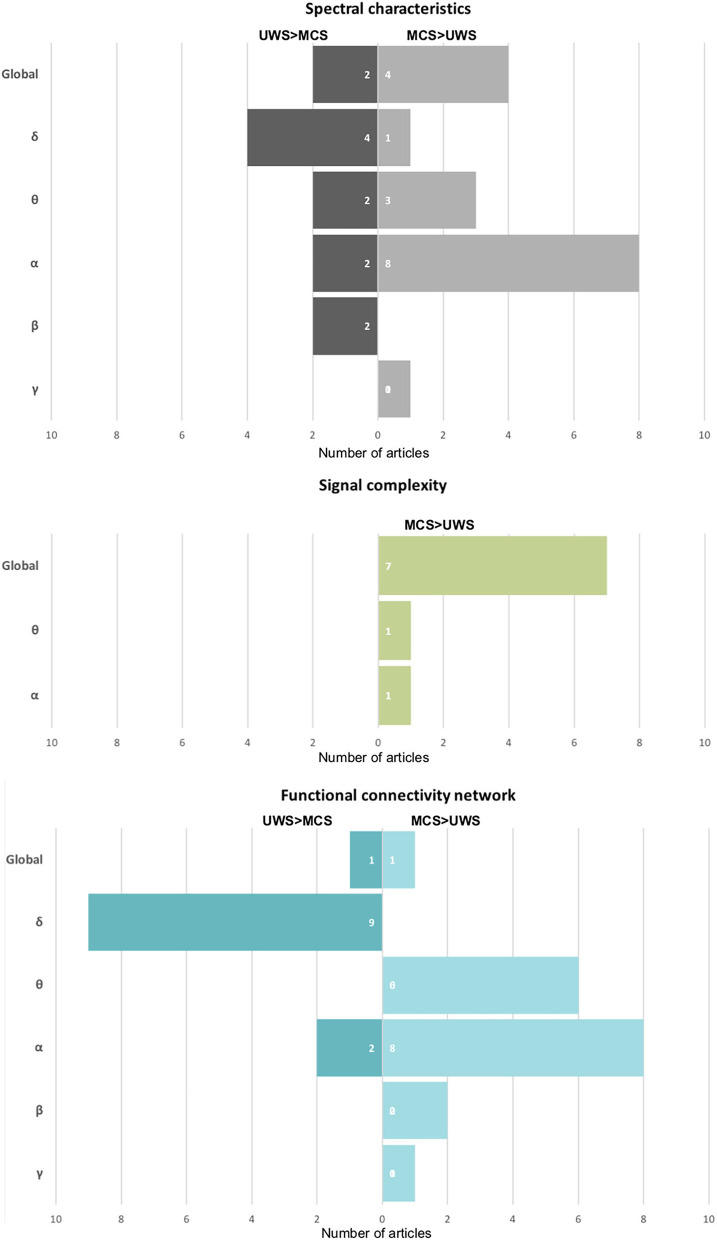
The summary of the number of publications shows a particular effect on spectral characteristics, signal complexity, and functional connectivity networks. On the left side of the chart: a stronger effect in UWS in comparison to MCS, on the right one—opposite—a stronger effect in MCS than in patients with UWS.

One of the most popular methods used to assess oscillatory brain activity is the analysis of power spectrum density. The power of brain activity was reduced overall in patients with DoC compared with healthy controls and those with severe neurological cognitive damage (Leon-Carrion et al., 2008; Babiloni et al., 2009; Naro et al., 2016, 2018). A more detailed analysis showed an increase in slower band power (δ, θ) and a prominent drop-off in higher bands (α, β) in the patient group, so the decrease of power is not uniform (Chennu et al., 2014; van den Brink et al., 2018), refer to Table 1. The power of faster oscillations is significantly reduced in patients with DoC (Coleman et al., 2005; Babiloni et al., 2009; Chennu et al., 2014; Naro et al., 2018; Stefan et al., 2018). In the case of patients with UWS, in comparison with those with MCS, brain activity is dominated by the low oscillation of the δ band (Lehembre et al., 2012a; Höller et al., 2014; Sitt et al., 2014; Naro et al., 2016, 2018; Piarulli et al., 2016; Stefan et al., 2018; Lutkenhoff et al., 2019) (refer to Figure 2). A significant increase in the δ band in patients with UWS in comparison with MCS and healthy controls was observed mainly in frontal areas (Lehembre et al., 2012a; Naro et al., 2016). As noted by Chennu et al. (2014) and Naro et al. (2018), about 80% of the overall spectral power was concentrated within the lowest EEG band, suggesting a severe impairment of cortico-thalamo connections (Gloor et al., 1977).

In turn, patients with MCS were usually characterized by stronger activity in higher bands (θ, α, β) and weaker in the δ band compared with UWS (Lechinger et al., 2013; Sitt et al., 2014; Naro et al., 2016, 2018; Lei et al., 2022). Thibaut et al. (2021) showed similar differences between patients with MCS* and UWS. In particular, the presence of an α band in the parietal areas seems to be crucial for consciousness (Babiloni et al., 2009; Lehembre et al., 2012a; Sitt et al., 2014; Naro et al., 2016) (refer to Figure 2). As shown by Babiloni et al. (2009), in the case of patients with UWS who had not recovered, the α band in the posterior areas was extremely weak, which seemed to be a reliable predictor of a bad outcome.

The results mentioned above are in line with the clinical observations based on visual inspection of the EEG signal of patients with DoC. Schiff (2016) proposed a theoretical framework to assess the integrity of thalamo-cortical and cortico-cortical networks. According to this theory, a higher level of anterior forebrain corticothalamic integrity is correlated with a higher level of consciousness, independent of the degree of injury to other brain areas (Schiff, 2016; Forgacs et al., 2017, 2020). The condition of thalamo-cortical integrity after severe brain injury is reflected in four different EEG regimes assessed *via* the signal spectrum related to predominant frequency power: A type—mostly δ activity (below 4 Hz); B type-θ activity (5–7 Hz); C type-θ, and β (15–40 Hz) activity; D type-α (8–12 Hz) and β activity. Based on a model “ABCD” spectral pattern, “A” indicates complete loss of corticothalamic integrity; “D” indicates full recovery of corticothalamic integrity; while “B” and “C” represent interim regimes with distinct physiological foundations (Schiff, 2016; Forgacs et al., 2017). The low oscillations of δ activity, uniform over the scalp, observed in patients with UWS correspond to an “A” type EEG regime and are interpreted as corticothalamic deafferentation—caused either by the impairment of synaptic transmission (van Putten and Hofmeijer, 2016) or by white matter lesions (Gloor et al., 1977). Regarding patients with MCS, the vast majority of studies showed stronger θ activity in MCS in comparison with UWS. This seems to be related to the preservation of a certain amount of afferent input to cortical neurons, which corresponds to “B” and “C” type EEG regimes. Such a hypothesis confirms stronger θ activity in patients with MCS in comparison to severe neurocognitive disorders (SND), which suggests that loss of consciousness is related to deeper brain injury, a low level of afferent input to cortical neurons, and stronger spontaneous oscillation of Layer V pyramidal cells. In comparison with patients with UWS, MCS brain activity is characterized by stronger α band oscillations that reflect normal tonic firing, and seem to be related to preserved corticothalamic integrity but at a lower level than in the controls (refer to Table 1).

## 4. Signal Complexity in Patients With DoC

The concept of entropy, first proposed as a thermodynamic principle, is now used widely to quantify the effects of anesthetic drugs on brain activity. EEG signal entropy methods may be based on time series (e.g., Approximate Entropy) and phase space analysis (e.g., Phase Lag Entropy), or on the frequency spectrum (e.g., Spectral Entropy) (Bandt and Pompe, 2002). Although these algorithms are sensitive to different properties of the signal, such as spectral bandwidth and amplitude distribution, and are generally difficult to interpret, they tend to have lower values if the signal slows down and becomes less complex (Ferenets et al., 2006). Different measures of entropy were used to assess differences in signal complexity among patients with DoC (Table 1). In most of the studies, these methods show a coherent picture—the global EEG signal complexity was lower in patients with UWS than in those with MCS (Sarà and Pistoia, 2010; Gosseries et al., 2011; Sarà et al., 2011; Wu et al., 2011b; Sitt et al., 2014; Stefan et al., 2018; Lee et al., 2019a) (refer to Figure 2). In particular, patients with MCS were characterized by more complex interactions between the activity of brain regions (Lee et al., 2019a), and by higher variability and periodicity of spectral entropy over time (Piarulli et al., 2016). As shown by Lei et al. (2022) in the longitudinal scheme, different entropy measures (Approximate Entropy, Sample Entropy, and Lempel-Ziv complexity) increased non-monotonically with the inflection point when the patients transferred from UWS to MCS. These results suggest that the transition from unconscious to conscious is not a linear phenomenon.

The best discrimination between UWS and conscious patient groups was obtained in θ (Sitt et al., 2014) and α bands (Stefan et al., 2018) (refer to Table 1). Moreover, lower values of complexity measures were observed in many studies on the effects of anesthetics (Liang et al., 2015) and sleep (Bruce et al., 2009) during the loss of consciousness.

## 5. Changes in Network Functional Connectivity in Patients With DoC

Brain function relies on the co-activation of several structures in functional networks (Damoiseaux et al., 2006). Some of them (i.e., the default mode network, DMN) seem to be related to the mechanisms of consciousness (Mason et al., 2007; Boly et al., 2008; Boveroux et al., 2010). While the complexity of brain networks is indirectly reflected by entropy measures as discussed in the previous section, the most detailed information about the functioning of the brain is provided by an examination of network functional connections. In the case of the EEG signal, there are many approaches to the assessment of this phenomenon, which have been applied to assess brain function in patients with DoC (for review refer to Bai et al., 2017; Corchs et al., 2019; Table 1). In the following subsection, we turn our attention to network functional connectivity in UWS and MCS.

### 5.1. Changes in Network Functional Connectivity in Patients With UWS

Considered at a global level, patients with UWS exhibit general deficits in information sharing, observed mainly over central and posterior areas and across medium-to-long distances (King et al., 2013). This has been interpreted as possibly related to disrupted thalamo-cortical and cortico-cortical interactions due to diffuse white matter lesions common in patients with persistent UWS and hypoactivation of the DMN, encompassing the precuneus and posterior cingulate cortex located over centro-parietal regions. This interpretation was supported by fMRI studies (Vanhaudenhuyse et al., 2009; Demertzi et al., 2015). Moreover, the analysis of particular bands showed more detailed, specific functional connectivity patterns related to the preserved level of consciousness. The most common pattern in patients with UWS, as compared with MCS and healthy controls, contains a decrease in the number of functional connections in higher bands (mostly α and θ) and stronger communication in the δ band-similar to the spectral power in these bands, as described in Section 3 (Höller et al., 2014; Sitt et al., 2014). Reduced functional connectivity patterns in frequencies higher than δ (mostly α and θ) in patients with UWS, compared with MCS, have been revealed in many studies (Lehembre et al., 2012a; Cavinato et al., 2014; Sitt et al., 2014; Chennu et al., 2017; Naro et al., 2018; van den Brink et al., 2018) (refer to Figure 2 and Table 1). The most significant impairment of information flow was observed over fronto-posterior areas (Lehembre et al., 2012a) in both θ (Lehembre et al., 2012a) and α (Cavinato et al., 2014; Chennu et al., 2017), which are presumably related to the preserved consciousness (Sitt et al., 2014; Chennu et al., 2017) (discussed in the next Section 5.2).

In the study of Höller et al. (2014), patients with UWS showed increased long-distance functional connectivity between frontal and parietal/occipital regions not only in θ, but, surprisingly, also in β frequencies, similar to the findings of Stefan et al. (2018), who reported stronger global coherence in the β band. It is difficult to interpret the preserved functional connectivity in higher bands in these patients, which is commonly linked with some functional networks observed in the resting state (Brookes et al., 2011). However, it should be noted that the EEG signal of patients with DoC has unusual characteristics and may be contaminated by massive artifacts, especially in the higher frequency range.

### 5.2. Changes in Network Functional Connectivity in Patients With MCS

Similar to healthy individuals, patients with MCS reveal the presence of functional connectivity networks, especially fronto-posterior (Bai et al., 2021; Naro et al., 2021), at higher frequencies. However, the strength of these connections was on average weaker in comparison with healthy controls (Fingelkurts et al., 2012; Chennu et al., 2014, 2017; Schorr et al., 2016) and patients with the severe neurocognitive disorder (Leon-Carrion et al., 2012). These results were also convergent with those of Chennu et al. (2014), who reported stronger functional connections in α and weaker connections in δ in healthy controls. In turn, the strength of fronto-parietal α connections was correlated with the level of preserved consciousness (from MCS-, MCS+, eMCS, locked-in syndrome, to controls) (Chennu et al., 2017). Moreover, functional networks in the α band were more pronounced on the local and global level in conscious controls than in patients with DoC (Chennu et al., 2017). However, some studies report preserved functional connectivity not only in α, but also in two other bands (θ, β) as a neural marker of consciousness in MCS (Lehembre et al., 2012a; Höller et al., 2014; Sitt et al., 2014; Naro et al., 2018). The stronger connectivity in θ and α bands is interpreted as the most important characteristic of the functional networks of conscious individuals (Lehembre et al., 2012a; Höller et al., 2014; Sitt et al., 2014). Regarding functional connections in θ, two interpretations are possible: on the one hand, increased coherence in this band is observed in patients who have suffered structural brain injuries over the entire scalp (Schiff et al., 2014). This is in line with the observation of stronger functional connectivity measures in the θ band observed in patients with DoC in general, in comparison with controls (Chennu et al., 2014; van den Brink et al., 2018). On the other hand, increased connectivity in θ and α in MCS compared to UWS occurs over mesio-parietal areas and may be related to the key role of fronto-parietal networks in a “global workspace” in generating conscious states, and it may, therefore, be expected that their activity is disrupted in patients with UWS (Sitt et al., 2014). These results are in line with those obtained by Bai et al. (2021), who showed reduced functional connectivity in patients with DoC in comparison to HC, especially fronto-parietal connections in α band. Moreover, they described four key transient brain states preserved in patients with DoC: anterior, posterior, sensorimotor, and visual. The dynamics of switching between these states were different in patients with UWS and MCS: the formers spent more time in the anterior state, while the latter—in sensorimotor, visual, and posterior ones. Moreover, the transition between the sensory and posterior states was suppressed in patients with UWS as compared to MCS. Among those with MCS, two subgroups of patients are distinguished: Patients with MCS− showing volitional reactions such as object localization and visual pursuit, and patients with MCS+ reacting to commands. A detailed examination of brain connections in these two subgroups was conducted by Chennu et al. (2017), who observed similar α connectivity in patients with MCS− and MCS+, but, in the case of the latter group, they seemed to be situated over the frontal and parietal areas. Importantly, the strength of frontal, central, and parietal functional connections was related to brain metabolism as measured by positron emission tomography. Moreover, patients with MCS− connectivity in the δ band was much stronger than in MCS+, which suggests a lower degree of cortical deafferentation in patients showing high-level behavioral responses. Similarly, the stronger connectivity in α and weaker in δ band was observed in patients with MCS* in comparison to UWS. On the other hand, patients with MCS had stronger connectivity in θ band than MCS*, which is in line with the previous hypotheses on the role of particular EEG bands in mechanisms of consciousness. To summarize, the functional connectivity patterns of patients with MCS are characterized by preserved structure at higher frequencies. In comparison with healthy controls, however, their strength is reduced. This seems to be in line with the fMRI results that show that the strength of correlation in DMN is related to the level of consciousness (Vanhaudenhuyse et al., 2009).

## 6. Resting-state EEG Measures in Sleep and After Anesthetic Administration

The patterns of brain activity described in patients with DoC corresponds to the characteristics of brain activity in loss of consciousness in other contexts. The increase in low frequency bands, observed particularly in patients with UWS, has been reported in other cases of loss or alteration of consciousness: during deep anesthesia (Steriade et al., 1993; Purdon et al., 2013), temporal lobe seizure (Englot et al., 2010), and slow wave sleep (Timofeev et al., 2001), seen as functional deafferentation. In numerous studies, the increase of low frequency power was associated with anesthetic administration and loss of consciousness, e.g., during induction with sevoflurane and propofol in combination with remifentanil, there is a gradual increase in θ and δ bands in anterior areas, which spreads to posterior ones (Gugino et al., 2001). As isoflurane concentration increases, the power spectrum below 4 Hz strengthens (Hagihira, 2015). In turn, Purdon et al. (2013) and Banks et al. (2020) reported the propofol-induced increase of δ and α bands in anterior regions. Similarly, stronger δ-range activity was reported during a complex-partial seizure (Englot et al., 2010), accompanied by a decrease in cerebral blood flow in the bilateral frontal and parietal cortices, than during a temporal lobe seizure with preserved consciousness (Blumenfeld et al., 2004). The EEG signal of patients with UWS is characterized by lower values of complexity measures than in MCS and healthy controls. Similar levels of complexity and regularity of brain activity were observed in deep anesthesia (Liang et al., 2015; Shin et al., 2020) and during sleep (Burioka et al., 2005; Bruce et al., 2009). The comparison of 12 different entropy algorithms applied to EEG data registered in anesthesia induced by gamma-aminobutyric acid-ergic agents (isoflurane or sevoflurane anesthesia) showed in each case a similar effect of decreasing entropy measures as anesthesia deepened, which then increased during recovery (Liang et al., 2015). With higher doses of anesthetic drugs, the EEG signals becomes more regular and its frequency pattern simplifies. In turn, the analysis of regularity and spectral content of EEG signal during sleep showed lower values of the entropy measures in relation to slowing brain activity during the subsequent non-rapid eye movement (NREM) stages and gradual loosening of consciousness (Burioka et al., 2005), which was negatively correlated with the power of the δ band (Bruce et al., 2009).

The vast majority of studies indicate stronger δ functional connections in UWS than in MCS and the presence of higher frequency bands in patients with MCS, which are crucial for preserved consciousness. Such hypotheses are in line with the studies of Lee et al. (2019b), who showed emphasized phase-locking at low frequencies, particularly in posterior cortical areas, in conscious experiences during NREM sleep in comparison with the unconscious. These were interpreted as cortical bistability in thalamocortical circuits connected with loss of consciousness. Notably, the conscious experience was accompanied by higher phase-locked values in the α and β bands. The importance of δ band functional connectivity in loss of consciousness was indicated during propofol-induced sedation as well, demonstrating the lower level of responsiveness, stronger δ power, and local δ functional connectivity in parietal regions (Lee et al., 2017). On the other hand, a preserved pattern of α activity seems to be connected with consciousness (Lee et al., 2019b; Banks et al., 2020). Loss of consciousness during the N2 and N3 stages of NREM and under anesthesia was associated with α-band connectivity limited to anterior regions (Banks et al., 2020). In comparison, during wakefulness α-band functional connectivity was mainly observed within and between temporal and parietal regions. Interestingly, the authors obtained no differences in long-distance connections between conscious and unconscious conditions, such as were observed in patients with MCS in comparison with UWS.

## 7. Prognostic Value of Resting-State EEG Measures

Finally, some of the resting-state EEG measures have prognostic value for patients' long-term recovery. One of them is the power of α oscillations, which proved to be related to recovery during follow-up (Babiloni et al., 2009; Fingelkurts et al., 2011; O'Donnell et al., 2021). To identify other prognostic indicators of a favorable outcome for patients with DoC, an analysis of brain oscillatory microstates was conducted (Fingelkurts et al., 2011). Its results indicated that a bad outcome was related to an increased rigidity in brain activity, which was reflected by a lower diversity and variability of EEG signal, and a higher probability of occurrence of δ, as well as slow θ oscillations in non-survivors. Similarly, Stefan et al. (2018), Sarà and Pistoia (2010), and Sarà et al. (2011) showed that the complexity of the EEG signal allowed prediction of patients' outcomes; however, these results are not coherent, and the study of Gosseries et al. (2011) did not confirm this hypothesis. Regarding the functional networks, Schorr et al. (2016), Chennu et al. (2017), Bareham et al. (2018), and Bareham et al. (2020) demonstrated that the functional connectivity may also have a prognostic value. As shown by Bareham et al. (2018) in a longitudinal examination of four patients with DoC, the process of regaining consciousness was accompanied by the establishment of the functional connectivity in the α band and decreasing in the δ range. These results were partially confirmed 2 years later by Bareham et al. (2020) who indicated functional connectivity in the α band, the power of θ band, age of a patient, and arousal levels as the most important predictors of behavioral recovery. On the other hand, Chennu et al. (2017) observed a clear relationship between EEG-based δ connectivity and outcome—patients with negative outcomes had strong δ functional connections across large parts of the central and parietal areas. In turn, Schorr et al. (2016) indicated that fronto-parietal (in δ, θ, α and β) and parietal (in δ and θ) coherence could predict recovery from UWS, suggesting that both short and long-distance connections may have a prognostic value and are critical for the preservation of consciousness.

## 8. Summary and Conclusion

The extensive literature on the EEG-based resting state research in patients with DoC shows that the brain activity of patients with UWS is dominated by low δ oscillations, both in spectral power and the connectivity domain. The flow of information in higher bands is severely reduced and the variability of brain activity time course is much lower than in patients with MCS. Additionally, the brain functional networks of patients with UWS are characterized by a less complex structure. Patients with MCS are characterized by preserved functional connectivity patterns in higher frequencies—in comparison with patients with UWS, stronger activity in higher frequencies and weaker activity in the δ band are observed. It seems that patients with MCS have a more integral fronto-parietal network, and the overall pattern of connectivity, although weaker in higher frequencies, is more comparable to that of healthy controls. Moreover, the brain activity of patients with MCS is more complex and variable over time than that of patients with UWS.

The extensive literature on the EEG-based resting state research in patients with DoC shows quite coherent profiles of EEG functional connectivity in patients with UWS and MCS. However, some contrary observations are presented. This may be due to the different data analysis approaches used in this field, the use of quite small and highly heterogeneous experimental groups, and the low quality of recorded data. It seems that the greatest variability of the results obtained is related to functional network analysis. Generally, methods of functional connectivity can be classified into two groups—model-free approaches (e.g., coherence, phase lag index) or model-based approaches dependent on the autoregressive model (Granger family indices). They all rely on different internal parameters and are sensitive to various characteristics of measured signals, and none is optimal for all types of signals with different signal-to-noise ratios, or different types of artifacts (Wang et al., 2014). Some research groups have also implemented customized algorithms that are difficult to interpret and compare with more standardized methods. The implementation itself of each method also needs to be considered, because different mathematical or implementational nuances may yield different results.

The EEG signal registered in patients with DoC is very often contaminated by different artifacts to a much higher degree than is the case in healthy subjects. Despite efforts to clean these data manually and the use of automated toolboxes, the quality of the data still seems questionable. It appears that muscle activity in particular, which is very common and strong in these patients, can influence the results obtained and their discrepancies between particular studies in higher frequency ranges.

Moreover, a large number of measures related to functional brain networks have been reported to be important for consciousness mechanisms and require further examination, e.g., cross-frequency coupling (Naro et al., 2021), time-course characteristics of functional networks (Bai et al., 2021), or neural monitoring of visceral inputs (Candia-Rivera et al., 2021). Notably, the reviewed works are based on indicators (parameters) obtained from the EEG signal. However, new machine learning approaches are applied directly to the raw EEG signal (after filtering and artifacts rejection), showing successfully the most important features related to mechanisms of consciousness (Lee et al., 2022). Finally, another interesting avenue for further research is a combination of different techniques of neuroimaging for the examination of preserved connectivity in patients with DoC in a more complex manner, i.e., taking into account the presence of brain lesions or analysis of the patterns of functional networks obtained by fMRI.

## Author Contributions

AD-B, MZ, and KJ-S reviewed the articles and wrote the manuscript. All authors contributed to the article and approved the submitted version.

## Funding

This research was partially supported by the Polish National Science Centre (UMO-2018/31/B/ST7/01888) and a Grant from SWPS University of Social Sciences and Humanities SUB/IPsy/04/2021/04.

## Conflict of Interest

The authors declare that the research was conducted in the absence of any commercial or financial relationships that could be construed as a potential conflict of interest.

## Publisher's Note

All claims expressed in this article are solely those of the authors and do not necessarily represent those of their affiliated organizations, or those of the publisher, the editors and the reviewers. Any product that may be evaluated in this article, or claim that may be made by its manufacturer, is not guaranteed or endorsed by the publisher.
